# Cognitive, Behavioral and Educational Outcomes in Children Aged 5–11 Years With Spina Bifida in Northern Ireland

**DOI:** 10.1002/bdr2.2434

**Published:** 2025-01-18

**Authors:** Yogesh Gopal Parajuli, Marlene Sinclair

**Affiliations:** ^1^ School of Nursing Ulster University Belfast Belfast Northern Ireland UK

**Keywords:** behavior, cognition, education, neurodevelopment, spina bifida

## Abstract

**Introduction:**

While improved medical and surgical care for children with pina bifida has improved their survival, some may have lower cognitive, behavioral and educational performance. The paper assesses the effect of spina bifida on cognitive, behavioral, and educational outcomes in 5–11 year olds.

**Methods:**

A cross‐sectional study design was used where data were collected from parents/guardians and teachers using Behavior Rating Inventory of Executive Function, second edition (BRIEF2), Strengths and Difficulties Questionnaire (SDQ), and Teacher Academic Attainment Scale (TAAS).

**Results:**

Nineteen parental and 13 teacher responses were received for children with spina bifida, and 8 parental and seven teacher responses for children without Spina Bifida. Overall, the majority of the sample were female. Children in both groups performed at a similar level across subscales of BRIEF2 with the exception of Working Memory. No group differences were found in SDQ scales as assessed by parents; teacher assessment of conduct problems. Hyperactivity/inattention and peer problems were higher for children with spina bifida. Anticipated matched analysis was not possible due to unequal number of participants between the groups. Children with spina bifida performed similarly as peers without spina bifida in all subjects across the curriculum with the exception of English, Mathematics, and History.

**Discussion:**

Based on this small sample, a potential need for evidence‐based interventions to assist children with spina bifida in the cognitive area of working memory and also in English, Mathematics and History is postulated. Larger longitudinal studies are required to confirm these findings.

## 1 Introduction

Neural tube defects (NTDs) relate to congenital defects in the development of the main organs of the central nervous system (CNS), namely the brain, spine, or spinal cord (Copp and Greene 2010). NTDs occur due to incomplete closure of the neural tube during embryonic development, which is normally completed within 30 days of conception (Jones, Jones, and del Campo 2013). The most commonly occurring NTDs are anencephaly and Spina Bifida, which occur in almost equal frequencies (Au, Ashley‐Koch, and Northrup 2010), although only Spina Bifida is compatible with survival (Liptak 2010; Liptak and El Samra 2010). The average worldwide prevalence is estimated to range from 1 to 10 cases per 1000 births (Imbard, Benoist, and Blom 2013).

All children need well‐developed cognitive and behavioral skills to interact with the world in a positive way and achieve various goals in life (Armstrong 2018; Bong 2012; Darling‐Hammond and Bransford 2007). While concerns regarding the long‐term outcomes for children with Spina Bifida have been raised previously (Ewing‐Cobbs, Barnes, and Fletcher 2003), these have been highlighted by the increasing rate of survival among these children (Fletcher and Brei 2010; Patel et al. 2019; Shin et al. 2012). It is important to recognize that the social and educational needs of individuals with spina bifida are pivotal to help inform evidence‐based service delivery and preparation for adulthood (Woodhouse 2008). Evidence‐based social care and interventions have been shown to be effective for improving intellectual development in children with CNS disorders like spina bifida (Blair and Raver 2014; Bruder 2010; Guralnick 1997, 2005; Ramey and Haskins 1981; Robinson et al. 2014; Wenar and Kerig 2000). At present, there is a gap in our understanding of how spina bifida affect cognitive, behavioral and educational outcomes among children with spina bifida. The skills associated with the cognitive and behavioral domains and educational achievement are vital for functional independence. Therefore, these outcomes should be better understood when individuals with spina bifida are at an early age (Blair and Raver 2016; Heffelfinger et al. 2008; Sawin 2017; Stubberud et al. 2014, 2015). Shortfalls in these outcomes may help for referral to appropriate early intervention systems for evidence‐based action. Timely support for any delays or difficulties in these three domains may help to optimize the potential in their later grades in which poses higher demands for learning including speed and organizational ability and be more independent in their later life (Blair and Raver 2016; Diamond and Ling 2016).

The purpose of this study was to explore to what extent there are differences across the cognitive, behavioral, and educational outcomes among children with spina bifida compared with other children without this condition. It will allow for the development of theory to understand the setbacks that children with spina bifida experience at early stage of development. Through identifying these hurdles, researchers and practitioners can design and implement interventions that enable growth for this target population. Northern Ireland was a suitable country in which to conduct this research because it has the highest rate of live births with spina bifida in the United Kingdom and Western Europe enabling potential access to a large sample of children (Public Health Agency (PHA) 2015; European Surveillance of Congenital Anomalies—EUROCAT 2015).

### 1.1 Aim

The aim of the study was to investigate the effect of spina bifida on cognitive, behavioral, and educational outcomes in 5–11‐year‐olds in Northern Ireland.

## 2 Methods

### 2.1 Participants

Participants in the study were children with spina bifida and their peers from the same class. Children with spina bifida were recruited through Shine (Spina Bifida Hydrocephalus Information Networking Equality), which provides specialist support to anyone living with spina bifida and/or hydrocephalus throughout NI. Shine identified children with spina bifida aged 5–11 years from among those registered in the Shine database and alive at the time of the study. A total of 70 children were identified as potential participants, and it was decided to invite all their parents/guardians to participate in the study. Shine agreed to contact parent/guardian of each eligible child when the approval was received from the ethics committees overseeing this study. Two ethics committees associated with this study provided clearance: Ulster University's Institute of Nursing and Health Research (INHR) Governance Filter Committee and the South West—Exeter Research Ethics Committee (REC reference: 18/SW/0004). Upon receiving consent from parents/guardians, booklets containing assessment tools for assessing child cognitive and behavioral outcomes were sent in stamped addressed envelopes.

The children without spina bifida studying in the same class as the children with spina bifida were also included in the study (joint total, *N* = 27). The school Principals were asked to choose one child each without spina bifida who closely matched the child with spina bifida in terms of similar date of birth, same gender, and, additionally, only for those children in special schools, having a physical disability other than spina bifida. Teachers completed a booklet each for the child with spina bifida and without spina bifida. Children whose parent/guardian could not understand English were not included in the study.

### 2.2 Measuring Outcomes

There are two types developmental screening tools recognized to be used: (i) directly administered by professionals from pathway of care and (ii) completed by parent/primary caretaker/teacher of the child. Although, directly administered tools are more comprehensive they take longer time to complete, and could be more suitable to serve as a follow‐up to the screening test report completed by parent/primary caretaker/teacher indicating any difficulties in the child (Glascoe and Marks 2011; Glascoe 2005; Hamilton 2006). Collaboration with parents/guardians and teachers using tools with systematic closed questions has been reported to be effective, competent, and inexpensive to detect shortfalls in child development as accurately as health professionals (Glascoe 2003, 2015; Glascoe and Marks 2011; Rydz et al. 2005). Another advantage of these tools is that they engage parents/guardian and teachers as active participants in the child's development as well as helping to enable their relationship with people engaged in pathway of care for children (Hamilton 2006). Therefore, the study considered inclusion of parents/guardians and teachers for assessment of the outcomes in children and a systematic search for the suitable standard tools to use.

### 2.3 Cognitive Outcomes

The Behavior Rating Inventory of Executive Function, second edition (BRIEF2) (Isquith, Roth, and Gioia 2014) was selected to measure cognitive outcomes. The BRIEF instruments have been used to study populations of children diagnosed with varying developmental disorders, such as autism spectrum disorder (ASD), attention deficit hyperactivity disorder (ADHD), language disorders, apart from the factors such as premature birth that may trigger such disorders, as well as developmental conditions (Gioia et al. 2000, 2015) including those with spina bifida (Burmeister et al. 2005; Rague et al. 2022; Zabel et al. 2011).

#### 2.3.1 Scoring of BRIEF2

Parents/guardians and teachers respond to each item on the BRIEF2 questionnaire by circling the appropriate answer, with 1 corresponding to Never (N), 2 corresponding to Sometimes (S), and 3 corresponding to Often (O)—based on their observation of child over the past 6 months. The score for each response was transferred to the scoring sheet for summation to give the total raw score for each clinical scale and index in BRIEF2. Once the raw scores were calculated for the BRIEF2 scales and indexes, the *T* score for each raw score from the parent/guardian form and the teacher form was identified using the appropriate normative table by gender and the four developmental age groups given in the BRIEF2 manual. *T* scores were used for the interpretation of the level of child's performance as rated by parents/guardians and teachers on the BRIEF2 forms. A score above 60 is reflective of modest difficulties and a score above 70 suggests more significant concerns. These *T* scores are linear transformations of the raw scale scores (mean = 50, standard deviation = 10) and provide information about an individual's scores relative to the scores of respondents in the BRIEF2 standardization sample (Gioia et al. 2015).

### 2.4 Behavioral Outcomes

The strengths and difficulties questionnaire (SDQ) (Goodman 1997) was used for assessing behavioral outcomes. The SDQ is a short behavioral screening tool used for children aged 3–16 years. It contains 25 questions to be completed by the parent/guardian or teacher based on their observation of child over the past 6 months. The rating is done on a three‐point scale (“not true,” “somewhat true,” and “certainly true”). Each of the subscales scores range from 0 to 10, although a higher score is indicative of deeper problems for all subscales, except for the prosocial scale which is reverse scored in that higher scores correspond to lower difficulties in prosocial behavior. The use of same items and scales for parents and teachers, together with the ready availability of the scale contributed to its utilization in the present study.

### 2.5 Educational Outcomes

Although the data for key stage (KS) 1 and 2 are maintained by teachers in all mainstream schools in NI to record each pupil's scale score in English and mathematics, it is recognized that special schools might deviate from the national curriculum and so may not have KS 1 and KS 2 data. Therefore, the teacher academic attainment scale (TAAS) (Johnson, Marlow, and Wolke 2011) was chosen as a useful tool to conduct a uniform screening for the educational performance of children regardless of which type of school they are attending. TAAS is completed by the teacher, who rates each child relative to the average level of performance expected of his/her class. It contains eight items rating a child's educational performance on a 5‐point scale (“very below average,” “below average,” “average,” “above average,” “very above average”).

### 2.6 Socio‐Demographic Measures

A short questionnaire was included in the booklet such as the age of the mother at the time of birth, the highest level of education achieved by the mother, child's area of deprivation (Northern Ireland Multiple Deprivation Measures [Northern Ireland Statistics and Research Agency 2017]), and any additional support the child was receiving in school. The database of postcodes within each super output area (SOA) and the 2017 NIMDM rankings were accessed after accepting the terms and conditions of Northern Ireland Statistics and Research Agency (NISRA), Belfast.

### 2.7 Statistical Analysis

The data obtained from parents/guardians and teachers were entered into the Statistical Package for Stata version 24 for analysis. Descriptive statistics in the form of mean age and frequency tables were used to summarize the area of deprivation (quintile). The children's BRIEF2 and SDQ scores were compared using an independent samples *t* test. The TAAS scores of children with spina bifida were compared with the performance of children without spina bifida using a chi‐square test after creating two categories: (1) below average and (2) average and above average. Potentially concerns about shortfalls in the child's development were identified based on the categorization of scores following the manual for BRIEF2 and SDQ.

In order to evaluate the influence of predictors on the developmental scores of children, crude and adjusted analyses were conducted. Crude analyses demonstrate the total effect of the predictor on the outcome variable. Adjusted analyses aim to account for potential confounding factors, showing a potential direct effect of the predictor on the outcome. There were four covariates known to affect the outcome studied were considered one at a time, on a priori basis in separate regression models; age of child, child's area of deprivation, mother's education level and mother's age at the birth of child. Although demographic factors can be theorized as more distal, they were included as initial predictors in the model to evaluate their associations with the child's cognitive, behavioral, and educational outcomes (Black and Krishnakumar 1998; Bukodi and Goldthorpe 2013; Duncan et al. 2018; Masten and Coatsworth 1998).

The continuous outcomes (score by parents and teachers) were analyzed using linear regression models with robust standard error estimates. The robust standard errors were used to account for the higher similarity between children that were part of the same class and rated by the same teacher. Crude and regression analyses allowing robust standard errors were performed. For the crude analysis, only the outcome and the group of which the child was part of (with/without spina bifida) were included in the model. For adjusted analyses, the following covariates were included in separate models: child age, child's area of deprivation, maternal education and maternal age at birth resulting in four models for parent/guardian data and four models for teacher data. Adjusted models control for potential confounding effects of the covariates on the association between the exposure and outcomes.

Considering the small sample size in our study, robust standard error estimation was adopted for all analyses conducted.

### 2.8 Linear Regression Model Assumptions

*Symmetric distribution of the residuals*: The symmetrical distribution of the residuals confirmed the symmetrical distribution of the dependent variables (BRIEF2 GEC scores and SDQ total difficulties scores). The symmetry of residuals was checked by visual inspection of their histogram.

*Independence of observations*: Each parent/guardian had only one child in the sample, and each child received an independent score. However, as children from the group of children without spina bifida were selected/rated by teachers in the same class as a child with spina bifida, analyses should ideally be matched. It was not possible to obtain a 1:1 matched pair between children with/without spina bifida due to the different number of participants in each group (see details in Section 3) and to the limited sample size, thus all analyses were performed without matching.

The lack of independence between teacher observations could increase the number of false positives (i.e., generate spurious statistically significant results between children with spina bifida and children without spina bifida, where they do not really exist) (McDonald 2009). The regression analyses were performed using robust standard errors to correctly estimate the standard errors, especially for those that were assessed by the same teacher and were part of the same class. It was not possible to perform multilevel analyses (Baayen, Davidson, and Bates 2008; Mumper 2017) due to the very small sample size.

*Homoscedasticity*: In the standard regression model, the assumption of homoscedasticity of the regression error term is made, that is its variance is assumed to be constant in the population, conditional on the explanatory variables (Kirkwood and Sterne 2010).

## 3 Results

### 3.1 Socio‐Demographic Characteristics

Socio‐demographic information for all children who took part in the study and their parents/guardians is presented in Table 1. Inclusion of children in the study was dependent on the response from their parents/guardians Principals and teachers. At first, 70 parents/guardians of children with Spina Bifida identified through Shine were invited to participate in the study, of which 20 responded, and 19 completed booklets, giving a response rate of 27.14%. Twenty School principals were identified from those 20 parental responses and contacted, of which 14 responded (with permission to contact teachers and also invite parents/guardians of children without spina bifida they selected), giving a response rate of 70%. Of the 14 parents/guardians of children without spina bifida who were invited to participate in the study through the school Principals, eight completed the booklets, giving a response rate of 57.14%. Teachers completed the booklets for 13 children with spina bifida (13/14 Principals' approval; 92.9% response rate) and 6 children without spina bifida (6/8 parents' approval; 75% response rate) (Table 1).

**TABLE 1:** Socio‐demographic characteristics of study sample and parents/guardians.

Characteristic	With spina bifida	Without spina bifida	*p*
	(*n* = 19)	(*n* = 8)	
Age of child (years)			
Mean	7.84	7.88	0.967
Standard deviation (SD)	1.74	2.1	
Range	5–11	6–11	
Gender			
Male (number [%])	6 (31.6)	1 (12.5)	
Female (number [%])	13 (68.4)	7 (87.5)	0.633
Tools completed by:			
Mother number (%)	19 (100)	8 (100)	
Area of deprivation			
Mean (standard deviation)	466.16 (252.74)	516.5 (210.9)	0.652
Range	33–877	20–713	
Quintile of MDM 2017 score			
Quintile 1 (most deprived)	4 (21.1)	1 (12.5)	0.710
Quintile 2	2 (10.5)	0 (0)	
Quintile 3	4 (21.1)	1 (12.5)	
Quintile 4	7 (36.8)	5 (62.5)	
Quintile 5 (least deprived)	2 (10.5)	1 (12.5)	
Maternal age			
Mean	31.05	28.25	0.252
Standard deviation (SD)	6.33	3.41	
Range (years)	21–44	23–33	
Maternal education			
Attended school (number [%])	13 (68.4)	3 (37.5)	0.206
University degree (Number [%])	6 (31.6)	5 (62.5)	

Most children in both groups of children with and without spina bifida were female. All the parents/guardians who completed booklets for both groups of children were mothers. There were no significant differences found in any demographic characteristic measured between children with and without spina bifida, or between their mothers using an independent sample *t* test for continuous variables and Fisher's exact test for categorical variables. For children with spina bifida, the mean maternal age at birth was 31 years. Mothers of children with spina bifida were slightly older than mothers of children without spina bifida, but this was not significant. Almost two‐thirds of the mothers of the children without spina bifida had obtained a university degree, compared with almost one third of mothers of children with spina bifida, although the difference was not significant.

Only two children with spina bifida in this sample attended special school, whereas, there were no children without spina bifida in the study from special schools. All children with spina bifida were receiving additional support from a classroom assistant while none of the children without spina bifida received any additional support. The information on type of spina bifida is not available as the plan to collect medical data from the trust was abandoned in view of low participation by parents/guardians in the study.

### 3.2 Outcomes

#### 3.2.1 BRIEF2 Parents/Guardians and Teachers Report

Table 2 presents the mean scores for BRIEF2 scales, index scores and the GEC from the parent/guardian and teacher ratings on the children with spina bifida and children without spina bifida who participated in the study. Children with spina bifida only had significantly higher mean scores on the BRIEF2 scales Initiate and Working Memory, and indexes CRI and the GEC than children without spina bifida (*α* > 0.05). After Bonferroni correction (*α* = 0.0038), this difference remains significant for the scale Working Memory only. With the exception of BRIEF2 scales, Emotional Control and Organization of materials and the BRI index, the teacher ratings for BRIEF2 were consistently higher on all scales for children with spina bifida compared with those without spina bifida. After the Bonferroni correction, the *p* value remained significant for the scales shift, initiate, working memory, planorganize, and task monitor, and for the indexes ERI, CRI, and the GEC (Table 2).

**TABLE 2:** Group comparison of the BRIEF2 scale, indexes, and the GEC *T* scores (parent/guardian and teacher rating).

Scale/index/composite measure	Parent rating			Teacher rating		
	Spina bifida	Without spina bifida	*p*	Spina bifida	Without spina bifida	*p*
	(*n* = 19)	(*n* = 8)		(*n* = 13)	(*n* = 6)	
	*T* score mean (SD)	*T* score mean (SD)		*T* score mean (SD)	*T* score mean (SD)	
Inhibit	53.11 (12.23)	51.13 (13.33)	0.711	54.54 (12.87)	45.00 (3.29)	0.025
Self‐monitor	53.05 (12.5)	47.13 (11.95)	0.247	60.31 (16.37)	45.5 (5.61)	0.028
Behavior regulation index (BRI)	53.47 (13.22)	49.63 (13.37)	0.312	57.62 (15.06)	45.00 (4.29)	0.064
Shift	63.05 (15.7)	52 (9.46)	0.077	62.92 (11.69)	43.67 (2.88)	< 0.001*
Emotional control	62.58 (12.5)	56.50 (14.77)	0.284	54.08 (13.55)	45.5 (3.21)	0.126
Emotion regulation index (ERI)	64.26 (15.08)	55.38 (12.65)	0.157	59.62 (11.28)	44.00 (1.9)	< 0.001*
Initiate	59.74 (11.66)	49.25 (10.66)	0.039	64.00 (10.97)	42.0 (1.55)	< 0.001*
Working memory	66.74 (11.14)	50.50 (11.36)	0.002*	70.69 (11.71)	43.67 (2.94)	< 0.001*
Plan/organize	57.74 (13.45)	51 (11.12)	0.225	60.69 (12.46)	42.00 (3.46)	< 0.001*
Task‐monitor	59.00 (10.93)	51.63 (7.58)	0.096	63.69 (12.66)	43.83 (2.86)	< 0.001*
Organization of materials	54.42 (11.65)	49.88 (8.17)	0.364	58.69 (18.45)	44.67 (3.62)	0.055
Cognitive regulation index (CRI)	61.68 (13.17)	51.13 (10.70)	0.031	65.54 (12.87)	42.00 (1.79)	< 0.001*
Global executive composite (GEC)	62.42 (13.77)	52.13 (11.34)	0.043	63.85 (12.75)	42.33 (2.25)	< 0.001*
SDQ						
Emotional symptoms	4.11 (2.81)	3.88 (2.85)	0.944	2.85 (3.44)	0.33 (0.82)	0.096
Conduct problems	2.16 (2.3)	1.63 (2.2)	0.365	1.38 (1.61)	0.00 (0.00)	0.033
Hyperactivity/inattention	5.37 (1.9)	4 (2.14)	0.084	5.62 (2.99)	1.50 (1.38)	0.013
Peer problems	2 (2.16)	1 (1.41)	0.271	3.00 (1.47)	0.17 (0.41)	0.001*
Prosocial behavior	8.21 (1.69)	9.13 (0.84)	0.250	7.31 (2.66)	8.83 (2.04)	0.208
Total difficulties	13.63 (6.47)	10.50 (6.7)	0.970	12.85 (6.40)	2.00 (1.27)	0.001*

* Bonferroni correction: *α* = 0.0038 (BRIEF2 scores); *α* = 0.01 (SDQ scores).

#### 3.2.2 SDQ Parents/Guardians and Teachers Report

Table 2 also presents the SDQ rating. There were no significant differences in the mean scores for children with and without spina bifida in the parent/guardian rating for any SDQ scale. By contrast, the teacher SDQ rating showed significantly raised mean scores for children with spina bifida compared with children without spina bifida on the SDQ subscales conduct problems, hyperactivity/inattention, and peer problems and in the SDQ total difficulty score (*α* < 0.05). The difference remained significant for the SDQ scale peer problems and the SDQ total difficulties score after the Bonferroni correction (*α* = 0.01).

#### 3.2.3 Linear Regression: Parent/Guardian and Teacher Rating of BRIEF2 GEC Score

Table 3 describes the crude and adjusted analyses of the mean difference of both BRIEF2 GEC scores and SDQ total difficulties scores between children with spina bifida and children without spina bifida from their parent/guardian's and teacher's ratings (linear regression). The *R* ^2^ for parent's assessments explained from 12.2% to 25.3% of the variance of the outcome (BRIEF2 GEC scores), while for teachers the percentage explained ranged from 50.7% to 56.3%. The *R* ^2^ indicates the quality of the models that were fitted; hence it is possible to note that outcomes measured from teachers were better explained by the predictors analyzed. Adjusting for the covariates did not decrease the estimates for either parents or teachers. Since all results in Table 3 refer to the same outcome, having a similar scale, the unstandardized beta coefficients are displayed. They represent on average how big, or small, the BRIEF2 GEC scores for children with spina bifida compared with children without spina bifida. The crude analysis showed a 10.3 (95% CI = −0.09; 20.70) score for parental rating—but without statistical significance between children with and without spina bifida. Apart from the crude analysis, four adjusted models were also fitted, adjusting for child's age, area of deprivation, maternal education, and maternal age at birth of child. Adjusted models control for potential confounding effects of the covariates on the association between the exposure and outcomes.

**TABLE 3:** Crude and adjusted linear regression models for parent/guardian's and teacher's ratings (BRIEF2 GEC and SDQ total difficulties scores).

Covariate	*R* ^2^	Unstandardised coefficient B	95% CI for B	*p*
Crude (BRIEF2 GEC score)				
Parent/guardian rating	0.122	10.3	−0.09 to 20.70	0.052
Teacher rating	0.507	22.17	13. 82 to 30.51	< 0.001*
Crude (SDQ total difficulties score)				
Parent/guardian rating	0.049	3.13	−2.53 to 8.79	0.265
Teacher rating	0.502	10.56	6.63 to 14.49	< 0.001*
Model 1 (BRIEF2 GEC score): Adjusted for child's age				
Parent/guardian rating	0.158	10.34	0.34 to 20.35	0.043
Teacher rating	0.532	21.81	12.83 to 30.79	< 0.001*
Model 1 (SDQ total difficulties score): Adjusted for child's age				
Parent/guardian rating	0.071	3.15	−2.43 to 8.72	0.255
Teacher rating	0.505	10.44	5.91 to 14.97	< 0.001*
Model 2 (BRIEF2 GEC score): Adjusted for area of deprivation				
Parent/guardian rating	0.146	9.84	−0.31 to 19.98	0.057
Teacher rating	0.510	21.73	10.93 to 32.52	0.001*
Model 2 (SDQ total difficulties score): Adjusted for area of deprivation				
Parent/guardian rating	0.151	2.69	−2.48 to 7.86	0.294
Teacher rating	0.512	10.39	6.11 to 14.67	< 0.00*
Model 3 (BRIEF2 GEC score): Adjusted for maternal education				
Parent/guardian rating	0.253	10.30	−1.62 to 22.21	0.087
Teacher rating	0.518	22.77	9.73 to 35.82	0.002*
Model 3 (SDQ total difficulties score): Adjusted for maternal education				
Parent/guardian rating	0.117	3.17	−3.22 to 9.56	0.314
Teacher rating	0.505	10.56	4.99 to 16.13	0.001*
Model 4 (BRIEF2 GEC score): Adjusted for maternal age				
Parent/guardian rating	0.146	11.39	0.67 to 22.11	0.038*
Teacher rating	0.563	23.61	14.80 to 32.43	< 0.001*
Model 4 (SDQ total difficulties score): Adjusted for maternal age				
Parent/guardian rating	0.161	4.24	−1.38 to 9.86	0.133
Teacher rating	0.604	11.45	7.48 to 15.43	< 0.001*

*Note:* Due to the small sample size, dichotomizing the outcomes causes an increased loss of information and increases the probability of generating zero cell estimates. Additionally, the reduced number of individuals in some categories cause the odds ratio to be overestimated. Therefore, logistic regression was not carried out.

The crude analysis showed no difference on parental ratings between children with and without spina bifida. In contrast, after adjustment for child's age and maternal age a statistically significant difference between children with and without spina bifida was identified, being, respectively, 10.34 (95% CI = 0.34; 20.35) and 11.39 (95% CI; 0.67; 22.11) higher for cases compared with children without spina bifida.

Regarding teacher's ratings, the crude analysis showed that scores were 22.17 (95% CI; 13.82; 30.51) higher for children with spina bifida compared with children without spina bifida. After adjustment for child's age and area of deprivation, the scores slightly reduced to 21.81 and 21.73, respectively. In turn, adjusting the models for maternal education and age the scores increased to 22.77 and 23.61, respectively.

As for SDQ total difficulties scores‐according to parental assessments, there was no difference in the mean SDQ total difficulties scores between the two groups of children (all *p* values > 0.05). When teacher's assessments were considered, all models showed statistically significant differences between two groups of children. The crude analysis showed that scores were 10.56 points (95% CI; 6.63; 14.49) higher for children with spina bifida compared with children without spina bifida. After adjustment for child's age and area of deprivation, the scores slightly reduced to 10.44 and 10.39, respectively. The highest difference was perceived for the model adjusted by maternal age, where scores were, on average, 11.45 points higher for children with spina bifida compared with those without spina bifida (Table 3).

#### 3.2.4 TAAS Report

Table 4 presents the educational outcomes in children with spina bifida and children without spina bifida from the TAAS reports. TAAS is completed by the teacher, who rates each child relative to the average level of performance expected of his/her class.

**TABLE 4:** Academic attainment as reported by teachers using TAAS.

Category	Spina bifida	Without spina bifida	
	*n* (%)	*n* (%)	*p*
English/literacy (*N* = 13)			
Below average	9 (69.23)	0 (0)	0.011*
Average and above	4 (30.77)	6 (100)	
Mathematics (*N* = 13)			
Below average	9 (69.23)	0 (0)	0.011*
Average and above	4 (30.77)	6 (100)	
Art and design (*N* = 13)			
Below average	4 (30.77)	0 (0)	0.255
Average and above	9 (69.23)	6 (100)	
Geography (*N* = 10)			
Below average	5 (50)	0 (0)	0.093
Average and above	5 (50)	6 (100)	
History (*N* = 11)			
Below average	6 (54.55)	0 (0)	0.043*
Average and above	5 (45.45)	6 (100)	
I.T. (*N* = 12)			
Below average	3 (25)	0 (0)	0.515
Average and above	9 (75)	6 (100)	
Science (*N* = 10)			
Below average	5 (50)	0 (0)	0.093
Average and above	5 (50)	6 (100)	
Design and technology (*N* = 11)			
Below average	5 (45.45)	0 (0)	0.102
Average and above	6 (54.55)	6 (100)	

Most children with spina bifida performed as well as the average level of performance expected of his/her class in I.T. Art and Design, and Design and Technology. Half of the children scored as having either average or above average performance expected of his/her class in Geography and Science. However, children with spina bifida were below average in English, Mathematics, and History compared with children without spina bifida. Fisher's exact test also showed differences between the two groups in these subjects (Table 4).

## 4 Discussion

This study was designed to investigate the cognitive, behavioral, and educational outcomes in a sample of children with spina bifida and children without spina bifida aged 5–11 years attending mainstream schools and special schools in Northern Ireland. While outcomes for groups which had included both children and adolescents together have been reported in the literature previously, no study to date has focused on all three outcomes simultaneously, and no study in the review has focused specifically and exclusively on children of primary school age. The unique contribution of this study is its investigation of child cognitive, behavioral, and educational outcomes elucidated through the assessment by the day‐to‐day carers of the child (parents/guardians and teachers) in home and school settings, rather than through direct assessment (Isquith et al. 2005; Isquith, Roth, and Gioia 2014; Jurado and Roselli 2007).

First, the findings from the study demonstrated that children with spina bifida (*n* = 19) performed similarly to children without spina bifida (*n* = 8) on multiple subscales of BRIEF2 that measured cognitive outcomes except for working memory from both parental and teacher reports, which is very important for brief storage and management of the information required to accomplish complex cognitive tasks such as reading, learning, reasoning, and knowledge. No group differences were found in SDQ scales for behavioral outcomes in children as assessed by parents; however, teacher assessment of peer relationship problems and total difficulties scores were higher for children with spina bifida. It is possible that teachers reported greater difficulties in children with spina bBifida primarily because they have observed that children have a wide repertoire of skills and assess the outcomes for these children against this standard. In addition, the academic curriculum imposes cognitive, behavioral, and educational demands on children when delivered in the school environment and assessed by teachers. As for teacher rated academic achievement, children with spina bifida performed similarly on all subjects except for English, Mathematics, and History where significant differences were found (*p* = 0.011*; *p* = 0.011*, and *p* = 0.043*, respectively).

Cognitive ability can influence other areas of functioning such as behavior and educational skills (Yeates et al. 2009). The combined interaction of these skills plays a vital role in life success or for high school and college achievement. These achievements enable greater independence and improve children's quality of life when they reach adolescence and adulthood (Bruder 2010; Guralnick 2005; Zelazo, Blair, and Willoughby 2016). The results of the current study indicated that although some children with Spina Bifida experience more difficulties in all three outcomes–measured using BRIEF2, SDQ, and TAAS– the differences were not significant for clinically elevated levels (indicative of having risk of potential difficulties) for overall cognitive abilities and potential behavioral problems; more than 69% of the children performed as well as expected for the class average in most subjects.

Regarding BRIEF2 subscales, ratings by parent and teacher of children group are also consistent with Tarazi, Andrew Zabel, and Mark Mahone (2008) who found significant differences in the initiate and working memory subscales of the metacognition index between participants with MMC and hydrocephalus and a comparison group without this condition. Using the BRIEF scale, Tarazi, Andrew Zabel, and Mark Mahone (2008) demonstrated that children and adolescents with myelomeningocele and shunted hydrocephalus (MMH) can suffer from impaired executive control behaviors and lack of maturation. Compared with the comparison group, cognitive control among mixed group of children and adolescents with MMH was significantly lowered. Overall, Tarazi, Andrew Zabel, and Mark Mahone (2008) demonstrated that children and adolescents with MMH had higher BRIEF scores (meaning greater difficulties) based on teachers' ratings.

The findings from this study are also consistent with a seminal study from Brown et al. (2008) who reported significant differences in the BRIEF initiate and working memory subscales between children and adolescents aged 10–17 years with MMC and a control group. Brown et al.'s (2008) longitudinal study demonstrated that the MI average for the group of children with spina bifida was statistically different from the normative mean. In addition, Brown et al. (2008) indicated that the BRIEF subscales organization of materials, initiation, working memory, and plan/organize for children with spina bifida had higher *t* scores compared with those without the condition. There was a positive relationship between spina bifida MMC and parental distress, suggesting that these deficits are a source of stress for parents who have a child with spina bifida. This current study also found differences in working memory supporting the research by Boyer, Yeates, and Enrile (2006), who evaluated how processing speed influence working memory in children with MMC. Boyer, Yeates, and Enrile utilized range of scales including the Children version of Paced Auditory Serial Audition Test (CHIPASAT), Working memory and Information processing speed, Weschler Intelligence Scale for Children‐Third Edition (WISC‐III), and Wechsler Individual Achievement Test (WIAT) scales. Based on the findings, children with MMC and hydrocephalus had deficiencies in working speed memory and information processing.

Working memory might be a prominent area of difficulty among children with spina bifida than other clinical scales in BRIEF2. The finding supports the findings by Rose and Holmbeck (2007) who showed a significant difference in the outcomes for working memory. The teacher rating in this study has also shown significantly higher proportion of clinically elevated *T* scores for (≥ 70) for working memory. Similar results were reported by Tarazi, Andrew Zabel, and Mark Mahone (2008) in a group involving both children and adolescents for BRIEF working memory, initiate, plan/organize, and MI index subscales. Similar to the current study, Calhoun and Mayes (2005) found that the processing speed index IQ with spina bifida was low, which is the measure of speed of task completion or effortlessness. This study's findings are also supported by Vinck et al. (2010) who found that children with spina bifida have difficulties with fine motor impairment, cognitive, and impaired motor quality.

The findings from this current study also support previous research by Raghubar et al. (2015), who found that children with MMC had similar educational outcomes in terms of arithmetic but scored poorly on standardized, approximate arithmetic subjects compared to typically developed children. Raghubar et al. (2015) also found that children with MMC had significantly different performances in orientating attention compared to typically developed children, but executive and alerting attention was similar. In addition, Raghubar et al. (2015) suggested that verbal working memory and fine motor skills acted as mediators in the relationship between group type and arithmetic. The relation of the group to fluency in maths was also mediated by visual–spatial working memory and fine motor skills. Similar results were also reported by Rose and Holmbeck (2007) among adolescents with spina bifida who had difficulties with attention and numbers, focused attention and planning. Rose and Holmbeck (2007) demonstrated that when intellectual functioning is controlled in children with spina bifida, they show greater deficiency in executive and attention functioning. The authors also suggested that the children's executive and attention deficits are predictors of the presence of social adjustment difficulties. Rose and Holmbeck (2007) concluded that adolescents with spina bifida demonstrate clear attention impairment, which may negatively influence their educational outcomes and ability to socialize.

## 5 Limitations

The study does not benefit from individual matching because of the small number of matched children in the comparison group that could be causing bias. As the sample size was low, this increased the probability of making a Type II error and reduced the ability to find differences in mean scores for BRIEF2 and SDQ between the children with spina bifida and children without spina bifida. In addition, a larger sample size would have yielded more precise results compared with those currently presented in this study, and the chances for detecting smaller group differences would have increased.

## 6 Conclusion

As for conclusion, there were no differences in terms of children's executive skills such as planning, inhibitory control skills but the study identified possibility of difficulty with memory according to both the parent/guardian and teacher reports. This supports previous research with mixed group of children and adolescents with spina bifida reported on struggle with maintaining information short‐term and in juggling other tasks while memorizing new information.

There were no heightened issues with behavior, but there were group differences for peer problems and overall behavioral difficulty in the teacher reports. Regarding educational outcomes, this study found children with spina bifida were below average in English, Mathematics, and History compared to children without spina bifida as reported by the teacher. Future research involving greater sample of children with Spina Bifida including direct testing of the student's academic performance is essential to confirm these findings. It is reiterated that all findings of this study need to be taken with extreme caution.

### 6.1 Stakeholder Engagement

Upon the completion of this study, the summary of findings was shared with Shine and the parents/guardians of children with Spina Bifida and some feedback was collected regarding how to improve their participation in research studies. It was understood that researchers would benefit from reaching out to more as well as less autonomous children and families. Simultaneously, suggestions for future research topics and areas of interest were made. Some of these aspects referred to the development of spina bifida across age spans, and its diverse phenotypes—such as ones having hydrocephalus with shunt, and associated care demand.

## Conflicts of Interest

The authors declare no conflicts of interest.

## Data Availability

The data that support the findings of this study are available on request from the corresponding author. The data are not publicly available due to privacy or ethical restrictions.
